# Marine Bacterial Biopolymers, Cyanobacteria and Seaweed Biomasses as Soil Amendments to Enhance Soil Wetting Properties and Water Retention

**DOI:** 10.3390/polym18131585

**Published:** 2026-06-26

**Authors:** Waqas Ali, Elio Coppola, Rossana Marzaioli, Vincenzo Zammuto, Luigi Marfella, Marina Morabito, Concetta Gugliandolo, Giulia Maisto, Flora Angela Rutigliano

**Affiliations:** 1 Department of Environmental, Biological and Pharmaceutical Sciences and Technologies, University of Campania Luigi Vanvitelli, Via Vivaldi, n. 43, 81100 Caserta, Italy; elio.coppola@unicampania.it (E.C.); rossana.marzaioli@unicampania.it (R.M.); luigi.marfella@unicampania.it (L.M.); floraangela.rutigliano@unicampania.it (F.A.R.); 2 Department of Chemical, Biological, Pharmaceutical and Environmental Sciences, University of Messina, Viale F. Stagno D’Alcontres 31, 98166 Messina, Italy; vincenzo.zammuto@unime.it (V.Z.); morabitom@unime.it (M.M.); concetta.gugliandolo@unime.it (C.G.); 3 Research Centre for Extreme Environments and Extremophiles, University of Messina, Viale Ferdinando Stagno D’Alcontres 31, 98166 Messina, Italy; 4 Department of Biology, University of Naples Federico II, Via Cinthia, 80126 Naples, Italy; g.maisto@unina.it

**Keywords:** soil water loss, seaweed wetting properties, seaweed-based amendments, exopolysaccharides, biosurfactant

## Abstract

Soil water retention is a key factor in ecological processes regulating ecosystem stability and resilience under environmental stress. In this regard, marine-derived additives may provide sustainable strategies to enhance soil water dynamics. Here, novel biopolymers derived from thermophilic bacteria, including six exopolysaccharides (EPS1–EPS6) and four biosurfactants (BS1-BS4), and biomasses from seaweed (BM1–BM4) and marine cyanobacteria (BC1–BC2), were investigated for their wetting properties and soil water retention. Wetting properties, including reduction in contact angle (RCA) and atmospheric-air moisture uptake (AMU), were monitored for 36 h at constant temperature (30 °C). The effect on soil water retention was evaluated in terms of water loss of soil samples treated with two different concentrations (0.5 and 1% *w*/*w*) of either biopolymers or biomasses in a microcosm consisting of 10 g of soil and 10 mL of water, kept at a stable temperature of 22 °C for 200 h (until complete evaporation occurred). BC2 derived from *Leptolyngbya* sp. 43.3 was the best wetting agent (RCA = 39.44%), while the EPS4 produced by *Bacillus horneckiae* SBP3 was the best humectant agent (AMU = 179.63%). Soils amended with bacterial biopolymers (EPS4, EPS5, EPS6, BS1 and BS3), as well as biomasses derived from cyanobacteria BC2 and seaweed BM1–BM4, produced better improvement in soil water retention, with marked effects at the concentration of 1% *w*/*w*. The lipopeptide BS1 was the most effective in water loss reduction over a specific time of 96–125 h at both concentrations. These findings highlight the potential of these materials as nature-based solutions to improve soil-mediated ecosystem resilience to drought under climate change.

## 1. Introduction

Soil water has a major role in ecosystem functioning, regulating primary productivity, microbial activity, nutrient cycling, and plant–soil interactions across terrestrial ecosystems [1]. Recently, global climate change has extremely disturbed the rainfall sequence, amplifying drought events, resulting in the reduction in soil moisture, which implies a major threat to food security [2]. Prolonged drought conditions have significantly reduced crop yields by 20 to 30%, directly compromising soil ecosystem services provision [3]. Furthermore, recent findings underline that droughts have involved a 40% water loss, leading to a 25% reduction in crop yields (including citrus fruits, wheat, and vineyards) and causing economic losses estimated at €2.7 billion. This is particularly worrying for regions in Southern Europe where water reservoir levels are critically low; current volumes have decreased by 45% compared to 2023 [4,5] and 25% of municipalities have been forced to implement water-saving measures [6]. The limited availability of water can lead to the formation of a water-repellent barrier in soil, a phenomenon known as water repellency [7]. This barrier significantly reduces the rate and capacity of water absorption of soil, leading to increased runoff and a reduction in water available for crops [8]. Therefore, to face water repellency it is crucial to adopt such strategies that enhance soil’s capacity to retain water and to minimize water losses. These strategies are essential for improving soil health and ensuring sustainable crop production, particularly in regions under water scarcity. Adding organic amendments like manure, straw and biochar is a common strategy to reduce soil water loss [9]. These amendments increase soil organic carbon, which acts as a binding agent to stabilize soil structure, thereby enhancing permeability and conserving soil moisture [10,11]. While these amendments effectively enhance soil water retention, their cost remains significantly high [12,13].

Recently, the use of microbial biopolymers and seaweed-derived biomasses has attracted the attention of researchers as sustainable soil amendments to improve soil water retention [2,14]. Biopolymers are organic materials produced by different living organisms (green plants, animals, microbes, and fungi) as part of their life processes [15], which occur abundantly in nature [16]. Due to their biodegradability and minimal environmental impact, biopolymers present a promising option for the improvement of soil characteristics thanks to their capacity for acting as a biological “glue” within soil pores. When exposed to water, they swell and enlarge soil pores and alter their size distribution, favoring moisture storage [17,18]. As a result, the permeability enhances, facilitating the passage of water [19]. Similarly, biopolymers extracted from seaweed, including alginate and carrageenan, are most effective in addressing water repellency through the swelling mechanism [19]. Soil application of alginate at 1% has been shown to enhance water availability for crops [20,21], while carrageenan application at concentrations of 1–2% increased water absorption as a result of its high swelling properties and ability to gradually release water in soil [22].

Likewise, bacterial-based biopolymers (xanthan gum and gellan gum) are extensively applied in soil due to their numerous benefits, such as their ability to enhance soil’s water retention capacity by absorbing significant amounts of water, up to a thousand times their dry weight, and improve soil structure as well [23,24]. Xanthan gum at 0.5% and gellan gum at 1% have been shown to outperform in terms of water retention and plant survival under drought stress, likely due to the formation of a stable and well-structured topsoil layer. They acted as an emulsifier, which supports high water-holding capacity in the inner soil [25,26,27].

Several studies have explored the impact of various biopolymers, such as alginate, carrageenan, xanthan gum, and gellan gum, on soil water retention [20,21,22,28], while biopolymers, including exopolysaccharides, biosurfactants and biomasses (seaweed and cyanobacteria), have not yet been thoroughly investigated. In this framework, thermophilic bacteria, cyanobacteria and seaweed biomasses represent three biologically and ecologically distinct, yet complementary, sources of soil hydrating materials [19]. Thermophilic bacteria are heat tolerant and thrive under elevated temperature conditions; they can synthesize exopolysaccharides and biosurfactants with surface active, natural binding and wetting characteristics and can enhance soil particle aggregation and water distribution [29,30]. In addition, cyanobacterial biomass are photosynthetic prokaryotes that form cohesive biocrusts and secrete extracellular polymeric substances, which are crucial for soil structure stabilization and moisture retention [31]. Seaweed biomasses are multicellular macroalgae, naturally enriched in polysaccharides (e.g., alginates, carrageenans, ulvans) capable of forming hydrogels that improve soil aggregation, aeration and water holding capacity [32,33]. Despite their taxonomic and ecological differences, all three groups lead to the production of polymer-rich substances that could be used as a nature-based amendment for improving soil wettability and water retention. Therefore, this study aims to examine the effects of different biopolymer/biomass additions on soil water retention, evaluated in terms of soil water loss. Understanding their influence on soil water dynamics is instrumental to better understand their potential contribution to ecosystem resilience under climate-driven water stress. We hypothesized that these materials could enhance soil water retention and thereby support key ecological processes associated with soil functioning. The findings of this study may contribute to the development of sustainable and nature-based strategies for mitigating the impacts of climate change on terrestrial ecosystems. The expected results could broaden our knowledge of the effects of biopolymers/biomass application, while at the same time evaluating their potential use as a soil amendment.

## 2. Materials and Methods

### 2.1. Origin of Strains

Marine thermophilic bacteria were previously isolated from the shallow hydrothermal vents of the Aeolian Islands (Italy) [34,35] and characterized phenotypically and genotypically. In detail, *Geobacillus thermodenitrificans* B3-72 [36], *Bacillus licheniformis B3-15* [37], *Bacillus* sp. *B3-75* [38] and *Bacillus licheniformis* md 4-1-1 [38] originated from Vulcano Island, while *Bacillus horneckiae SBP3* DSM 103063 [39] and *Bacillus licheniformis* s7s-1 [38] were isolated from vents off Panarea Island.

Cyanobacteria were isolated from seawater samples collected from the coastal zone of the Strait of Messina (Italy). The filamentous strains Cyano 40 and Cyano 43.3, based on 16S rRNA sequencing, were phylogenetically attributed to *Kamptonema okenii* (similarity of 98%) and *Leptolyngbya* sp. (similarity of 95%), respectively. Marine thermophilic bacterial and cyanobacteria are part of the collection of the “Research Centre for Extreme Environments and Extremophiles” at the Department of Chemical, Biological, Pharmaceutical, and Environmental Sciences of the University of Messina (Italy). Strains are preserved for long-term storage at −80 °C in 40% (*v*/*v*) glycerol.

*Sargassum muticum* (Ochrophyta) was collected from the Lagoon of Venice (45°25′42.6″ N, 12°19′50.7″ E), whereas *Ulva ohnoi* (Chlorophyta), *Agardhiella subulata* (Rhodophyta) and *Chaetomorpha aerea* (Chlorophyta) were collected from the brackish Lake of Ganzirri, in the Oriented Natural Reserve of Cape Peloro Lagoon, Messina (Italy) (site coordinates: 38°15′28.8″ N, 15°26′29.3″ E; 38°15′31.0″ N, 15°36′49.5″ E and 38°26′11″ N, 15°62′72″ E, respectively) [40]. Seaweeds were identified through DNA barcoding, using selected barcode markers and sequences deposited in BOLD Systems: *S. muticum* (AA05681), *A. subulata* (AAC0053), *C. aerea* (GRAPP001-17) and *Ulva ohnoi* (GRAPP015-17). Seaweeds are housed at the Phycological Lab Herbarium, University of Messina, Italy (https://sweetgum.nybg.org/science/ih/herbarium-details/?irn=253162, accessed on 17 June 2026).

With regard to morphology, thermophilic bacteria of the genus Bacillus are morphologically characterized as rod-shaped bacteria that aerobically form refractile endospores [41]. The cells of cyanobacteria attributed to the genera Leptolyngbya and Kamptenema are filamentous [42]. Marine algae showed the typical morphology of their respective species: *U. ohnoi* with large, fragile, easily torn thalli often bearing microscopic marginal teeth; *A subulata* with terete thallus morphology and *C. gerea* characterized by unbranched and yellowish-green filaments.

### 2.2. Production of Marine Biopolymers and Biomasses

Exopolysaccharides (EPSs) from thermophilic bacteria were produced as reported previously [34]. Briefly, each strain was inoculated in a minimal medium containing 0.01% yeast extract and seawater supplemented with 5% *w*/*v* of sucrose (SWY + SAC). Flasks were incubated for each strain at an optimal growth temperature for 48 h under shaking conditions at 250 rpm. The culture of each strain was centrifuged at 8000× *g* for 10 min, and the cell-free supernatant (CFS) was obtained by filtering through a 0.2-µm-pore-size membrane (Biogenerica, Catania, Italy). To ensure that no cells were present in the filtrates, 100 µL was spread onto plates of Tryptic Soy Agar (Condalab, Madrid, Spain) and incubated at 45 °C for 24 h. To inactivate the enzymes responsible for the EPS degradation, each CFS was heated at 100 °C for 20 min. Each CFS was treated with an equal volume of cold absolute ethanol added dropwise under stirring in an ice bath, held at −20 °C overnight, and then centrifuged at 10,000× *g* for 30 min. The pellets were washed twice with ethanol, dissolved in hot water (80–90 °C), and dialyzed (6–8 kDa-cutoff membrane SpectraPor Standard Grade RC Membrane) first against tap water (for 48 h) and then distilled water (for 20 h), lyophilized, and weighed. Carbohydrate content was evaluated by the phenol–sulfuric acid method using glucose as the standard [43].

Biosurfactants (BSs) production from thermophilic bacteria was performed as reported by [44]. Briefly, each strain was grown aerobically in novel MGV medium containing 20 g of NaCl, 10 g of (NH_4_)_2_SO_4_, 0.5 g of MgSO_4_ 7H_2_O, 3 g of meat extract (Sigma Aldrich, Milan, Italy), 3 g of yeast extract (Oxoid), 8 g of K_2_HPO_4_, 2 g of KH_2_PO_4_, and 30 g of sucrose or glucose per liter and incubated at optimal growth temperature in a rotary shaker at 130 rpm for 48 h. To obtain CFS, each culture was centrifuged at 8000× *g* for 10 min. To extract crude surfactants, each CFS was acidified at a pH of 2.0 using 2N HCl and kept overnight at 4 °C. Each surfactant was dissolved into a chloroform–methanol mixture (2:1, *v*/*v*), and the organic layer was separated using a rotary evaporation process (Rotavapor R-300, BUCHI Italia S.r.l, Conaredo, Italy) to collect the concentrated biosurfactant and then lyophilized.

To obtain cyanobacterial biomasses (BCs), *Kamptonema okenii* 40 and *Leptolyngbya* sp 43.3 were inoculated (OD_690nm_ = 0.1) into a 5 L flask containing 1 L of BG11 medium [45] under continuous bubbling with air (sterilized through a 0.45 μm membrane filter), at a light intensity of 200 µmol-photons m^−2^ s^−1^, and at 25 °C, as reported previously [46]. Air flow was provided at a rate of 1.75 L/min. To recover the cyanobacterial biomass, each culture was centrifuged at 8000× *g* for 10 min, dried, and weighed. Collected seaweeds (BMs) were washed with sterile seawater, manually cleaned of epiphytes, and air-dried [40].

Exopolysaccharides (EPSs) and biosurfactants (BSs) from thermophilic bacteria and biomasses from cyanobacteria (BC) and seaweeds (BMs) used in this study are listed in Table 1.

### 2.3. Assessment of Wetting Properties of Biopolymers and Biomasses

The effects of different biopolymer/biomass solutions at increasing concentrations (from 0 to 10 mg/mL) on the wettability of hydrophobic surfaces were determined, in collaboration with researchers of University of Messina, using the sessile drop technique on a hydrophobic substrate (polystyrene) at 25 °C. An image was captured with a high-resolution camera and analyzed using the Image J software V 1.54P Drop Snake plugin to measure the contact angle on sessile drop images [47]. The measurements were repeated thrice, and the reduction in contact angle (RCA) was reported as a percentage (%).

The atmospheric-air moisture uptake (AMU) was evaluated as described by [48]. Each biopolymer/biomass (10 mg) samples were placed in Petri dishes and were dried in a sealed desiccator. To ensure high relative humidity, the bottom of the desiccator was filled with sterile tap water, and samples were incubated for 36 h at constant temperature (30 °C). The samples were weighed to evaluate the increasing weight due to humidity uptake.

### 2.4. Evaluation of Soil Water Retention

Microcosm experiments were conducted to evaluate the effects, after the addition of each biopolymer or biomass, on the water retention of loamy–sandy soil (53.6% sand, 32.7% silt and 13.7% clay; acidic (5.35), with no salinity problem (EC, 854 µS cm^−1^)), as reported in Table 2. Six exopolysaccharides (EPS1–EPS6), four biosurfactants (BS1–BS4), two biomasses (BC1, BC2) derived from cyanobacteria and four biomass seaweeds (BM1–BM4) were applied to soil. Each microcosm experiment included two biopolymer/biomass concentrations (0.5% and 1% *w*/*w*) according to previous studies [25,49,50] and a treatment without any biopolymers/biomass (C, control soil). Ten milliliters of distilled water were added to each plate containing 10 g of dry weight (with or without biopolymer/biomass). Each treatment was replicated five times. The experimental sets were maintained at a constant temperature of 22 °C for about 200 h (until complete evaporation occurred). The effects of biopolymers/biomasses were evaluated in terms of soil water loss using gravimetric methods taken at regular sampling intervals (1–2 times per day). To better assess the temporal dynamics of the biopolymer/biomass-treated soils, compared to the respective control, water losses were expressed as a percentage of loss with respect to their relative control [51,52]. Moreover, to compare water loss of all biopolymers/biomasses together, water loss at a point before the equilibrium state (96–125 h) for each biopolymer/biomass (expressed per hour) was calculated.

### 2.5. Statistical Analysis

Descriptive statistics (means and standard deviation) for all biopolymer/biomass treatments were applied for each considered incubation time. Before running parametric tests, all nonnormally distributed data were logarithmically (Log10) transformed to obtain normal distribution, with equal variances respectively checked, also after transformation, by Shapiro–Wilk and Leven tests [53]. One-way Analysis of Variance (ANOVA) and Completely Randomized Design (CRD) (on each time interval for each soil water retention experiment), followed by Tukey’s post hoc test, were applied to determine the significance of differences (*p* < 0.05) among treatments. Two-way ANOVA (followed, if required, by the Bonferroni test) was applied over a period ranging from 96–125 h (points before the equilibrium state) using biopolymer/biomass treatments (EPS1-EPS6; BS1-BS4; BC1 and BC2; BM1-BM4) and concentrations (0.5 and 1% *w*/*w*) as factors. Statistical analyses were carried out using Sigma Plot 14 (Sigma Stat, Jandel Scientific, San Rafael, CA, USA), with MS Excel used to draw the figures.

## 3. Results and Discussion

### 3.1. Effect of Biopolymers/Biomasses on Wetting Properties (RCA and AMU)

The wetting properties were generally affected by assayed biopolymers/biomasses (Table 3). Among exopolysaccharides (EPSs), the reduction in contact angle (RCA) ranged from 9 to 32%, compared to the control (=0). EPS2 showed the most RCA reduction (32%), followed by EPS1 (23.78%) and EPS6 (22.44%). In the case of biosurfactants, their high ability to lower RCA was shown by BS4 (37.89%), followed by BS1 (36.27%). RCA for biomass from seaweed (BM1-BM4) varied from 14.42 to 28.36%, with a higher value observed for BM2. Overall, in terms of cyanobacterial biomass, BC2 showed the highest RCA reduction (39.44%).

The most pronounced reduction in contact angle (RCA) in treatments involving BC2 and BS4 indicated their higher capacity with respect to enhancing the affinity of the soil surface for water. A lower contact angle generally indicates that the biopolymers reduced the surface tension or altered the soil particle surface, increasing its wettable capacity [54]. The main reason behind this behavior is due to the formation of biofilm/physical barriers, which changed the surface properties and increased wettability and hydrophilicity. Consequently, the interaction between the surface and water increases, resulting in lower RCA values [55,56].

The atmospheric air moisture uptake (AMU) significantly varied (Table 3) in response to the tested biopolymers/biomasses. For the exopolysaccharides (EPSs), values ranged from 22.33 to 179.63%, with the highest result obtained with EPS4 (179.63%), followed by EPS5 (46%) and EPS6 (34%). For biosurfactants, AMU ranged from 33.50–90.53%, with BS3 achieving the higher AMU value (90.53%), followed by BS1 (43.87%). Furthermore, among cyanobacterial biomasses, BC1 was the most active, reaching an AMU value of 90.53%. Biomasses from seaweed biomass showed AMU values ranging from 28.03 to 59.60%, with BM2 recording the higher value (59.60%), followed by BM4 (49.43%).

In comparison to all groups together, EPS4 were the most efficient humectants, with the highest AMU values and a moderate RCA reduction, while BS4 resulted in the opposite behavior, with the strongest RCA reduction and intermediate AMU. Cyanobacterial and seaweed biomasses showed mixed profiles, with BC2 behaving more similarly to BS4 in terms of RCA, respectively.

Overall, the different behaviors observed for exopolysaccharides, biosurfactants, cyanobacterial biomasses and seaweed biomasses in terms of reduction in contact angle and AMU can be interpreted in terms of their chemical and structural characteristics. Bacterial EPSs are typically high-molecular-weight heteropolymers rich in hydroxyl and carboxyl groups, which confer strong hydrophilicity and enable extensive hydrogen bonding with water; these features explain the very high AMU of EPS4 and, more generally, the pronounced humectant behavior of EPSs [7,57]. Unlike exopolysaccharides, lipopeptide biosurfactants synthesized by *Bacillus* species contain both hydrophilic and hydrophobic domains, enabling efficient surface activity and interfacial tension reduction [58]. However, their structural properties are less favorable for extensive water retention. Accordingly, BS4 and BS1 were highly effective in decreasing RCA but displayed only intermediate levels of AMU (Table 2). In addition, biomasses from cyanobacteria and seaweed both contain extracellular polymeric substances and cell-wall polysaccharides (e.g., sulfated polysaccharides in cyanobacteria, alginates and carrageenans in seaweeds) that are hydrophilic and can swell, supporting RCA and AMU [59,60].

### 3.2. Effect of Biopolymers/Biomasses on Soil Water Loss

**Figure 1:**
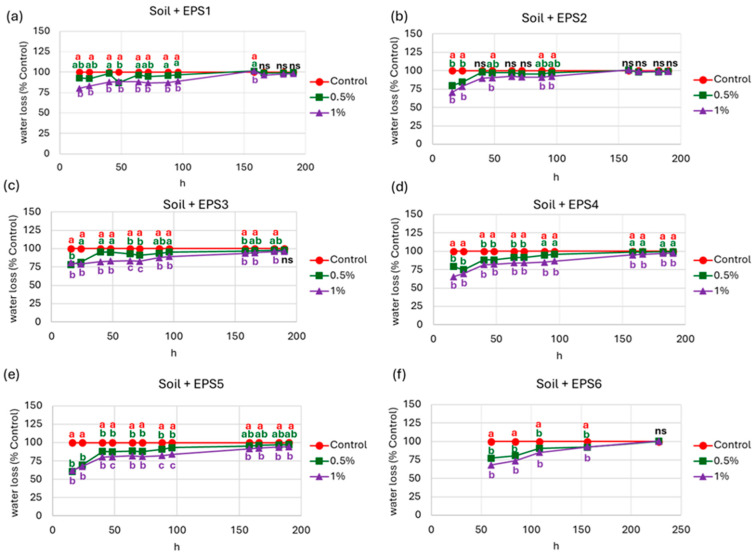
Values are the means (n = 5) of soil water loss (% of control) over time (h) in soil treated with exopolysaccharides: (**a**) EPS1, (**b**) EPS2, (**c**) EPS3, (**d**) EPS4, (**e**) EPS5 and (**f**) EPS6 at two concentrations (0.5% and 1% *w*/*w*) Different letters at each point indicate significant differences among treatments according to Tukey’s HSD test (*p* ≤ 0.05). Various colors of the letters correspond to the treatments—red = control; dark green = 0.5%; purple = 1%—while “ns” corresponds to non-significant differences between treatments.

**Figure 2:**
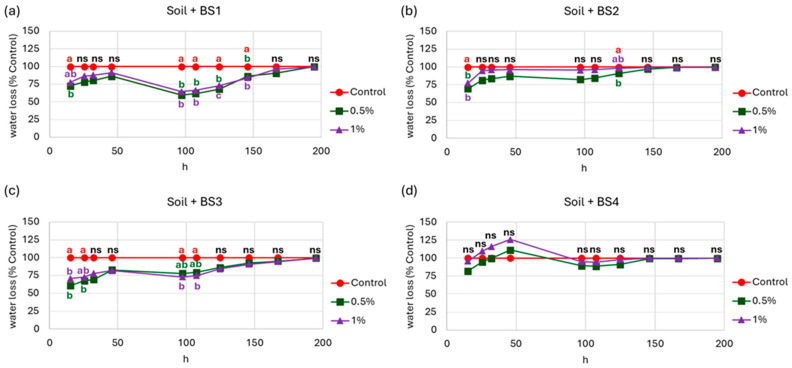
Values are the means (n = 5) of soil water loss (% of control) over time (h) in soil treated with biosurfactants: (**a**) BS1, (**b**) BS2, (**c**) BS3 and (**d**) BS4, at two concentrations (0.5% and 1% *w*/*w*). Different letters at each point indicate significant differences among treatments according to Tukey’s HSD test (*p* ≤ 0.05). Various colors of the letters correspond to the treatments—red = control; dark green = 0.5%; purple = 1%—while “ns” corresponds to non-significant differences between treatments.

**Figure 3:**
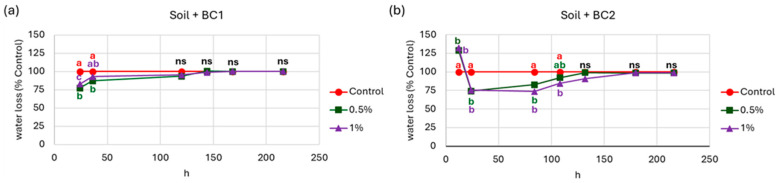
Values are the means (n = 5) of soil water loss (% of control) over time (h) in soil treated with cyanobacterial biomasses: (**a**) BC1 and (**b**) BC2 at two concentrations (0.5% and 1% *w*/*w*). Different letters at each point indicate significant differences among treatments according to Tukey’s HSD test (*p* ≤ 0.05). Various colors of the letters correspond to the treatments—red = control; dark green = 0.5%; purple = 1%—while “ns” corresponds to non-significant differences between treatments.

**Figure 4:**
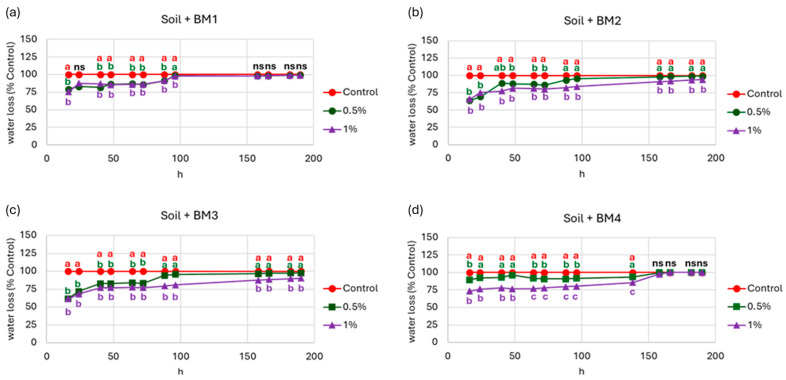
Values are the means (n = 5) of soil water loss (% of control) over time (h) in soil treated with seaweed biomasses: (**a**) BM1, (**b**) BM2, (**c**) BM3 and (**d**) BM4 at two concentrations (0.5% and 1% *w*/*w*). Different letters at each point indicate significant differences among treatments according to Tukey’s HSD test (*p* ≤ 0.05). Various colors of the letters correspond to the treatments—red = control; dark green = 0.5%; purple = 1%—while “ns” corresponds to non-significant differences between treatments.

Compared to the control, soil water loss was generally affected by the adding of biopolymers/biomass at each considered concentration (0.5 and 1% *w*/*w*; Figure 1, Figure 2, Figure 3 and Figure 4).

Exopolysaccharides (EPSs; Figure 1) had a significant effect on soil water loss (expressed as % of control) at both tested concentrations (0.5 and 1% *w*/*w*) compared to the control (=100). The lower water loss was recorded in response to the addition of EPSs (EPS1-EPS6), particularly at 1%, which remained significantly different from the control for up to 150 h of incubation (Figure 1). Only the samples treated with 1% EPS4 (Figure 1d) and 1% EPS5 (Figure 1e) maintained these differences compared to the control throughout all incubation periods. The 0.5% sample followed a similar trend but with less marked difference compared to the control. The observed improvements after EPS amendment could be due to the capacity of these biopolymers to act as hydrogels that hold water, directly promoting soil aggregate and the formation of water-trapping spaces [61]. These effects reduce water loss from the soil, providing a more consistent moisture supply for plants and microbes, especially under dry or drought-stressed conditions [62,63].

Furthermore, biosurfactant treatments (BSs; Figure 2) did not affect water loss (% of control) compared to the control (=100) in BS2 and BS4treated soils at both tested concentrations (0.5 and 1% *w*/*w*). BS3 at 1% significantly reduced water loss only during the mid transition stage, at nearly 90 to 110 h, compared to the control (Figure 2c). Moreover, BS1 (at both concentrations) significantly reduced water loss (Figure 2a) to 110 h compared to the control. The improved water retention is driven by the ability of biosurfactants to lower surface and interfacial tension, thereby enhancing water distribution and absorption within the soil [64,65]. The mechanism driving this improvement is the capacity of biosurfactants from *Bacillus licheniformis* (BS1) and *Bacillus horneckiae* (BS3) to reduce surface and interfacial tension [44]. By lowering these forces, the biosurfactants enhance water distribution and absorption throughout the soil matrix [43,44]. This was further confirmed by our findings in terms of a significant increase in AMU (Table 2); the higher AMU (45 and 90% in BS1 and BS3, respectively) may enhance soil wettability, facilitating water capture. This result confirms the role of these biosurfactants as wetting agents, consistent with previous studies that demonstrated their water-interactive properties on hydrophobic polystyrene surfaces [7,21].

Regarding the cyanobacterial biomass treatments (Figure 3), only BC2 at both (0.5 and 1% *w*/*w*) significantly improved soil water retention, maintaining a measurable effect for up to 110 h of incubation (Figure 3b). In contrast, BC1 treatments showed no significant impact on water retention and performed similarly to the untreated control, except at the initial stages of incubation. The effectiveness of the BC2 treatments can be attributed to the capacity of cyanobacteria to form a cohesive biocrust, which creates a protective surface layer that limits evaporation [66]. These findings aligned with [67], who observed that biological soil crusts developed from cyanobacteria acted as a stabilizing agent, stabilizing the soil surface and enhancing resistance to environmental stresses. This physical reconfiguration of the soil structure may also improve its hydraulic properties, as suggested by significant improvement in the moisture characteristic curve and overall water retention capacity that resulted from the inoculation of cyanobacteria in both loamy and sandy soils [68].

Treatment with seaweed biomass amendments (Figure 4) positively influenced soil water retention across all types (BM1−BM4) and concentrations compared to the control. BMs followed a similar trend as observed with EPS treatments (Figure 1), with the highest soil water retention recorded for the 1% addition. These treatments generally maintained a significant difference from the control for up to 150 h of incubation. Applications of BM2 (Figure 4b) and BM3 (Figure 4c) at 1% maintained these differences compared to the control throughout all incubation periods. These results may be attributed to the high content of polysaccharides, such as alginates, fucoidans, and carrageenans, inherent in seaweed biomasses. These polymers facilitate hydrogel formation, which enhances soil aggregation, aeration, and moisture retention [69,70], particularly in degraded soils [19].

**Figure 5:**
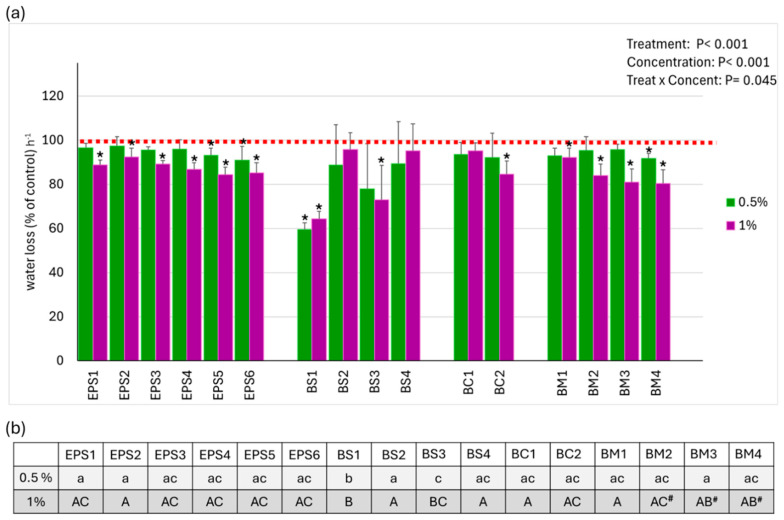
Values represent (**a**) means (n = 5; error bars indicate ± SD) of soil water loss (% of control) in response to biopolymers (EPS1–6 and BS1–4) and biomasses (BC1–2 and BM1–4) at two concentrations, 0.5% (green bars) and 1% *w*/*w* (purple bars), calculated over a 96–125 h period, just before the equilibrium state (no significant differences occurred). The red dashed line represents the untreated control (100%). Asterisks (*) indicate significant differences between treatments and control. Results of a two-way ANOVA (factors: treatment and concentration) are provided in the top-right panel. (**b**) Results of the Bonferroni post hoc test (*p* < 0.05) is presented by different lowercase letters, indicating significant differences corresponding to the 0.5% concentration, while different uppercase letters indicate significant differences corresponding to the 1% concentration. The hashtag (#) indicates significant differences between the two concentrations within the same treatment.

Furthermore, comparing all treatments by considering water loss at a point before the equilibrium state (96–125 h) for each biopolymer/biomass (expressed per hour) (Figure 5), each of them appeared more efficient in reducing water loss at the highest concentration (1%), apart from BS1, which was statistically significant compared to the control, at both concentrations (Figure 5a). The addition of BM2, BM3 and BM4 (marked with a hashtag, Figure 5b) was statistically different in a dose-depending manner. In summary, while most amendments improved soil water retention relative to the control, the degree of improvement depended strongly on the specific material, with the lipopeptide BS1 the most effective in terms of water loss reduction over a specific time of 96–125 h.

The incorporation of marine-based biopolymers or biomasses could increase water availability for plants and soil organisms in drought conditions [71].

When comparing groups and concentrations, EPSs (EPS3, EPS4 and EPS5) and BM3 at 1% *w*/*w* provided the largest and most persistent reductions in water loss over the entire incubation, whereas biosurfactants (BS1 and BS3) and cyanobacterial biomass BC2 mainly affected the mid-drying and early stages, respectively, reflecting their primary roles in enhancing wettability, infiltration and surface sealing rather than bulk water storage. The fact that BS1 remained effective at both 0.5 and 1% highlights that relatively low doses of biosurfactant are sufficient to modify soil hydraulic behavior, while EPSs and seaweed biomasses exhibit a stronger dose dependence, requiring higher concentrations to express their full hydrogel-like potential.

## 4. Conclusions

The addition of biopolymers and biomasses differently improved the soil hydrating properties, since their performance greatly depended on both their origin and different chemical structures. Specifically, BC2 derived from the cyanobacteria *Leptolyngbya* sp. 43.3 was the best wetting agent (RCA = 39%), and the EPS4 produced by *Bacillus horneckiae* SBP3 was the best humectant agent (AMU = 180%). In the case of water retention, all the EPSs reduced water loss, particularly at a 1% concentration, but EPS4 and EPS5 at 1% demonstrated higher soil water retention for the entire duration. Among BSs, BS1 was more pronounced in terms of retention of water at both concentrations (0.5 and 1% *w*/*w*) from 96 to 125 h. Regarding biomasses, BC2 derived from cyanobacteria significantly enhanced soil water retention at both concentrations (0.5 and 1% *w*/*w*), maintaining a measurable effect for up to 110 h of incubation. Seaweed biomasses (BM1−BM4) showed higher water retention only at 1% *w*/*w*. Moreover, a comparison across all treatments considering water loss at a point before the equilibrium state (96–125 h) indicated that each sample was efficient in reducing water loss at the highest concentration (1%), except for BS1, which demonstrated the highest efficiency at both tested concentrations (0.5% and 1% *w*/*w*). Overall, these results suggest that these treatments can retain water and could be a potential bio-based strategy to support soil water retention under drought conditions.

## 5. Implications for Field Conditions

Taken together, our findings provide proof of concept that marine-derived biopolymers and biomasses can alter soil wetting behavior and slow water loss under controlled conditions. On a larger scale, these materials will interact with a more heterogeneous pore network, including aggregates and macropores, so that hydrogels and wetting agents are likely to accumulate in specific zones (e.g., near the surface, along root channels or within fine pores), potentially enhancing infiltration in dry patches while having weaker effects in coarse domains. In cropped soils, roots will continuously modify pore geometry and act as dynamic sinks for water, so that improvements in retention may translate into deeper rooting, delayed onset of plant water stress or altered patterns of water uptake, rather than into a simple uniform reduction in evaporation. Temperature fluctuations and wet–dry cycles can also change the swelling, viscosity and degradation rate of polymeric materials, meaning that their impact on water retention may be stronger after recent application and gradually decline as they are transformed. Finally, resident microbial communities might incorporate these substrates into biofilms or extracellular matrices, reinforcing soil aggregation in some microsites while accelerating breakdown in others. For these reasons, field studies should not only monitor soil moisture and evaporation, but also examine changes in soil structure, root distribution and microbial activity to determine how the mechanisms identified in microcosms are expressed under realistic climatic and management conditions and whether they ultimately translate into improved crop performance or drought resilience.

## 6. Strength and Limitations

An important strength of this work is the integrated experimental design, in which highly diverse marine-derived materials were tested under identical conditions, allowing direct comparison of their effects on wetting properties and soil water loss. By jointly analyzing RCA, AMU and drying kinetics, we were able to identify function-specific best performers and to demonstrate that these biodegradable materials are promising with respect to their use as a soil amendment. Some limitations should be noted, the experiment was performed under controlled conditions and short time scales, without accounting for additional effects on soil properties (e.g., aggregate stability and pore network changes), plant responses, nutrient cycling, or potential trade-offs with other soil functions. Furthermore, we tested only two limited numbers of strains and species, so the generality of the observed patterns across soil types, climates and biopolymer formulations remain to be fully evaluated in future field trials.

## 7. Prospects

However, further studies are required to verify whether these positive effects persist over the long term, underlining the necessity of conducting pot and field-level assays to evaluate the effects on soil and plants. Moreover, further work should clarify how their physicochemical properties control soil hydraulic responses and evaluate their performance in combination with existing organic amendments and irrigation strategies, and their degradation dynamics, persistence under variable temperature and moisture regimes. Such studies, coupled with environmental and economic assessments, will be crucial to determine whether these marine-derived materials can be realistically integrated into climate-smart, nature-based soil management practices.

## Figures and Tables

**Table 1:** List of thermophilic bacteria, cyanobacteria, and seaweed with corresponding codes: exopolysaccharides (EPSs), biosurfactants (BSs), and biomasses (BMs/BC) used in this study.

Species/Origin	Code
	Biopolymers
Thermophilic bacteria	Exopolysaccharides
*Bacillus licheniformis* md 4-1-1	EPS1
*Bacillus* sp. B3-75	EPS2
*Bacillus licheniformis* B3-15	EPS3
*Bacillus horneckiae* SBP3	EPS4
*Bacillus licheniformis* sp. s7s-1	EPS5
*Geobacillus thermodenitrificans* B3-72	EPS6
	Biosurfactants
*Bacillus licheniformis* B3-15	BS1
*Geobacillus thermodenitrificans* B3-72	BS2
*Bacillus horneckiae* SBP3	BS3
*Bacillus licheniformis* s7s-1	BS4
	Biomasses
Cyanobacteria	
*Kamptonema okenii* 40	BC1
*Leptolyngbya* sp. 43.3	BC2
Seaweed	
*Sargassum muticum* (Lagoon of Venice)	BM1
*Ulva ohnoi* (Lagoon of Capo Peloro)	BM2
*Agardhiella subulata* (Lagoon of Capo Peloro)	BM3
*Chaetomorpha aerea* (Lagoon of Capo Peloro)	BM4

**Table 2:** Soil texture (by indicating sand, silt and clay percentage) and mean values (±standard deviation, n = 5) of bulk density (BD), pH, electrical conductivity (EC) and soil organic carbon (SOC) of the soil used for the experiment.

Soil Parameters	Value
Texture	loamy–sandy (53.6% sand, 32.7% silt and 13.7% clay)
Bulk Density (BD, g cm^−3^)	0.78 ± 0.10
pH	5.35 ± 0.08
Electrical Conductivity (EC, µS cm^−1^)	854.27 ± 115.79
Organic Carbon (SOC, g kg^−1^ d.w.)	22.34 ± 6.95

**Table 3:** Mean values (±standard deviation; n = 3) of reduction in contact angle (RCA) and atmospheric-air moisture uptake (AMU).

	RCA (%)	AMU (%)
Code	Control = 0	Control = 0
Exopolysaccharide		
EPS1	23.78 ± 0.09 b	22.33 ± 0.81 b
EPS2	32.00 ± 0.06 a	27.40 ± 0.68 b
EPS3	21.13 ± 0.32 c	25.63 ± 1.41 b
EPS4	9.00 ± 0.06 e	179.63 ± 12.60 a
EPS5	13.78 ± 0.96 d	45.87 ± 0.61 b
EPS6	22.44 ± 0.24 bc	33.83 ± 0.71 b
Significance	***	***
Biosurfactant		
BS1	36.27 ± 2.08 a	43.87 ± 1.39 b
BS2	25.20 ± 1.56 b	33.50 ± 2.05 b
BS3	29.78 ± 2.54 ab	90.53 ± 0.98 a
BS4	37.89 ± 1.01 a	43.23 ± 4.92 b
Significance	***	***
Cyanobacteria		
BC1	29.11 ± 1.68 a	90.53 ± 0.77 a
BC2	39.44 ± 1.96 b	27.40 ± 0.81 b
Significance	*	***
Seaweeds		
BM1	24.49 ± 1.53 a	40.73 ± 1.99 c
BM2	28.36 ± 0.98 a	59.60 ± 1.67 a
BM3	14.42 ± 0.84 b	28.03 ±1.39 d
BM4	18.27 ± 1.73 b	49.43 ± 1.56 b
Significance	***	***

Results of one-way ANOVA (* *p* < 0.05; *** *p* < 0.001) are reported in the last row within each biopolymer/biomass group and Tukey’s post hoc test results are shown with different letters.

## Data Availability

The original contributions presented in this study are included in the article. Further inquiries can be directed to the corresponding author.
